# Impact of simulated reduced injected dose on the assessment of amyloid PET scans

**DOI:** 10.1007/s00259-023-06481-0

**Published:** 2023-10-28

**Authors:** Peter Young, Fiona Heeman, Jan Axelsson, Lyduine E. Collij, Anne Hitzel, Amirhossein Sanaat, Aida Niñerola-Baizan, Andrés Perissinotti, Mark Lubberink, Giovanni B. Frisoni, Habib Zaidi, Frederik Barkhof, Gill Farrar, Suzanne Baker, Juan Domingo Gispert, Valentina Garibotto, Anna Rieckmann, Michael Schöll, on behalf of the AMYPAD consortium

**Affiliations:** 1 Wallenberg Centre for Molecular and Translational Medicine, University of Gothenburg, Gothenburg, Sweden; 2 Department of Psychiatry and Neurochemistry, Institute of Physiology and Neuroscience, University of Gothenburg, Gothenburg, Sweden; 3 Department of Radiology and Nuclear Medicine, Amsterdam UMC, Vrije Universiteit Amsterdam, Amsterdam, The Netherlands; 4 Amsterdam Neuroscience, Neurodegeneration, Amsterdam, The Netherlands; 5 Department of Radiation Sciences, Radiation Physics, Umeå University, Umeå, Sweden; 6 Clinical Memory Research Unit, Department of Clinical Sciences, Lund University, Malmö, Sweden; 7 Department of Nuclear Medicine, Toulouse University Hospital, Toulouse, France; 8 Division of Nuclear Medicine and Molecular Imaging, Geneva University Hospital, Geneva, Switzerland; 9 Nuclear Medicine Department, Hospital Clínic Barcelona, Barcelona, Spain; 10 Biomedical Research Networking Centre of Bioengineering, Biomaterials and Nanomedicine (CIBER-BBN), ISCIII, Barcelona, Spain; 11 Nuclear Medicine and PET, Department of Surgical Sciences, Uppsala University, Uppsala, Sweden; 12 Laboratory of Neuroimaging of Aging (LANVIE), University of Geneva, Geneva, Switzerland; 13 Geneva Memory Center, Department of Rehabilitation and Geriatrics, Geneva University Hospitals, Geneva, Switzerland; 14 Geneva University Neurocenter, Geneva University, Geneva, Switzerland; 15 Department of Nuclear Medicine and Molecular Imaging, University of Groningen, University Medical Center Groningen, Groningen, Netherlands; 16 Department of Nuclear Medicine, University of Southern Denmark, Odense, Denmark; 17 UCL Institute of Neurology, London, UK; 18 GE Healthcare, Amersham, UK; 19 Helen Wills Neuroscience Institute, University of California Berkeley, Berkeley, USA; 20 Molecular Biophysics and Integrated Bioimaging, Lawrence Berkeley National Laboratory, Berkeley, United States; 21 Barcelona βeta Brain Research Center (BBRC), Pasqual Maragall Foundation, Barcelona, Spain; 22 Centro de Investigación Biomédica en Red Bioingeniería, Biomateriales y Nanomedicina, Madrid, Spain; 23 Hospital del Mar Medical Research Institute (IMIM), Barcelona, Spain; 24 Universitat Pompeu Fabra, Barcelona, Spain; 25 Division of Nuclear Medicine and Molecular Imaging, University Hospitals of Geneva; NIMTLab; Center for Biomedical Imaging (CIBM), University of Geneva, Geneva, Switzerland; 26 Institute for Psychology, Universität Der Bundeswehr München, Neubiberg, Germany; 27 Department of Neurodegenerative Disease, UCL Queen Square Institute of Neurology, University College London, London, UK; 28 Department of Clinical Physiology, Sahlgrenska University Hospital, Gothenburg, Sweden

**Keywords:** PET, Dose reduction, Alzheimer’s disease, Neuroimaging, Amyloid

## Abstract

**Purpose:**

To investigate the impact of reduced injected doses on the quantitative and qualitative assessment of the amyloid PET tracers [^18^F]flutemetamol and [^18^F]florbetaben.

**Methods:**

Cognitively impaired and unimpaired individuals (*N* = 250, 36% Aβ-positive) were included and injected with [^18^F]flutemetamol (*N* = 175) or [^18^F]florbetaben (*N* = 75). PET scans were acquired in list-mode (90–110 min post-injection) and reduced-dose images were simulated to generate images of 75, 50, 25, 12.5 and 5% of the original injected dose. Images were reconstructed using vendor-provided reconstruction tools and visually assessed for Aβ-pathology. SUVRs were calculated for a global cortical and three smaller regions using a cerebellar cortex reference tissue, and Centiloid was computed. Absolute and percentage differences in SUVR and CL were calculated between dose levels, and the ability to discriminate between Aβ- and Aβ + scans was evaluated using ROC analyses. Finally, intra-reader agreement between the reduced dose and 100% images was evaluated.

**Results:**

At 5% injected dose, change in SUVR was 3.72% and 3.12%, with absolute change in Centiloid 3.35CL and 4.62CL, for [^18^F]flutemetamol and [^18^F]florbetaben, respectively. At 12.5% injected dose, percentage change in SUVR and absolute change in Centiloid were < 1.5%. AUCs for discriminating Aβ- from Aβ + scans were high (AUC ≥ 0.94) across dose levels, and visual assessment showed intra-reader agreement of > 80% for both tracers.

**Conclusion:**

This proof-of-concept study showed that for both [^18^F]flutemetamol and [^18^F]florbetaben, adequate quantitative and qualitative assessments can be obtained at 12.5% of the original injected dose. However, decisions to reduce the injected dose should be made considering the specific clinical or research circumstances.

**Supplementary Information:**

The online version contains supplementary material available at 10.1007/s00259-023-06481-0.

## Introduction

The introduction of amyloid-β (Aβ) tracers for positron emission tomography (PET) has enabled in vivo assessment of one of the earliest pathological markers of Alzheimer’s disease (AD). This has been a great step forward for the field of AD research, as it has allowed for extracting quantitative measures of disease state, measuring treatment effects, and differentiation between patients with AD or non-AD neurodegenerative disorders [1–6]. Three of the Fluorine-18-labelled amyloid PET tracers, [^18^F]flutemetamol, [^18^F]florbetapir and [^18^F]florbetaben, have been approved by the European Medicines Agency (EMA) and the Food and Drug Administration (FDA) and are now commonly used for visualising and quantifying Aβ pathology in clinical, research, and clinical trial settings.

Nonetheless, there are various challenges to the routine use of PET scans in these settings. First, PET examinations are expensive compared with commonly used imaging modalities, such as magnetic resonance imaging (MRI) and computed tomography (CT). A key contributing factor to these high costs is the complexity of the ligand synthesis and ligand availability [7]. Second, amyloid PET examinations have an associated radiation burden, typically between 6-7 mSv for Aβ tracers [8–10], and they are time-intensive, with acquisition protocols of 20 min in clinical routine and up to 110 min for research protocols [1, 11, 12].

Radiation burden in particular has been an obstacle when imaging healthy individuals and can restrict longitudinal and multi-tracer studies because of radiation safety regulations. This last aspect is of special importance given the current availability of, e.g., tau and synaptic density PET tracers that allow for characterizing different pathological aspects of AD [13, 14].

These issues emphasise the importance of investigating whether the currently recommended injected dose for PET examinations can be reduced without compromising diagnostic performance. The benefits of a reduced injected dose would be two-fold: first, it would reduce scanning costs and second, it would reduce radiation exposure for patients, study participants and staff. Investigating the effect of reduced injected doses also allows for indirectly assessing reduced acquisition time as they will scale with each other, although differences will occur due to small changes in tracer kinetics and detection events when using a reduced acquisition time as opposed to assessing reduced injected dose. In the context of AD, the possibility of having a shorter scan would be highly relevant given that patients with AD dementia are not always able to lay still in the scanner for the duration of the scan. Furthermore, shorter scans could also allow for increasing throughput.

PET scanners have constantly evolved in terms of hardware and software, leading to improved spatial resolution, sensitivity, and image quality [15–17]. Consequently, acquisition protocols should be reviewed regularly to determine whether any adjustments are warranted. For the FDA-approved [^18^F]flutemetamol tracer, recommendations regarding the injected dose have been set using images collected on older generation cameras such as the Siemens HR + scanner [18], and the same principle holds true for the [^18^F]florbetapir and [^18^F]florbetaben tracers. Previous studies have examined reduced injected doses for [^18^F]FDG, [^18^F]florbetapir and [^18^F]Genentech Tau Probe 1 (GTP1) in the context of AD. These studies demonstrated that it is feasible to reduce the injected dose, in some cases to as low as 10% of the original injected dose, without compromising the quantitative information extracted from the scan [19–21]. However, the implications of reduced doses on the clinical assessment of Aβ PET scans, remain largely unexplored. Furthermore, it has been suggested that visual assessment of reduced dose scans might be particularly challenging in grey-zone scans (i.e., scans displaying intermediate levels of Aβ pathology) [19].

The most straightforward method for evaluating the feasibility of a reduced dose is to scan a participant twice, once at the original dose and once at a reduced dose [21], however, this increases the participants' radiation burden. Additionally, biological variation between repeated acquisitions will increase uncertainty in the effect of reductions in dose. An alternative approach involves acquiring list-mode data and reconstructing or simulating a scan of reduced dose using a subset of these data [20, 22–26]. Currently, this method is not regularly employed because of increased processing complexity and large storage requirements for list-mode data. Finally, a third method is the use of bootstrapping to generate a larger set of low-dose images from a smaller dataset [19, 27]

In this study, we investigated the impact of reducing the injected dose in a research and clinical setting for [^18^F]flutemetamol and [^18^F]florbetaben by reconstructing previously acquired list-mode data that has been edited to simulate reductions in the injected dose. This method was chosen as it allows for a true simulation of a reduced dose, assessing the effect of a range of dose reductions and does not require additional scanning. More specifically, we investigated how reductions in the injected dose impact the standardised uptake value ratio (SUVR), Centiloid (CL) and the ability to visually assess PET scans with varying degrees of amyloid-β pathology. The overall goal of this project was thus to determine whether the vendor’s recommended injected dose could be reduced for [^18^F]flutemetamol and [^18^F]florbetaben, and to provide insight as to what dose reduction could be considered feasible.

## Methods

### Subjects and study protocol

In this study, 250 participants were retrospectively included. Of these, 175 participants were scanned with [^18^F]flutemetamol across two centres: Barcelonaβeta Brain Research Center (BBRC), Barcelona, Spain (*N* = 68), and Geneva University Hospital, Geneva, Switzerland (*N* = 107). Participants were part of the Amyloid Imaging to Prevent Alzheimer’s Disease (AMYPAD) Diagnostic and Patient Management Study (DPMS) [28] or Prognostic and Natural History Study (PNHS) [29] or were recruited from the Geneva Memory Clinic cohort (GMC). Another 75 participants were also part of the AMYPAD PNHS study, but scanned with [^18^F]florbetaben at the Centre Hospitalier Universitaire de Toulouse (CHUT), France. All PET data were available in list-mode format, as required per the design of the present study. Further cohort details can be found in Table 1. All participants had undergone standard neurological screening and neuropsychological assessment of each recruiting site. Prior to enrolling in the study, participants provided written informed consent in accordance with the Declaration of Helsinki and the International Conference on Harmonization Good Clinical Practice. Study protocols were approved by the local Medical Ethics Review Committees and can be accessed as part of their clinical trial registrations (EudraCT Number AMYPAD DPMS: 2017–002527-21 (2018–04-24) and AMYPAD PNHS is registered at www.clinicaltrialsregister.eu with EudraCT number AMYPAD PNHS: 2018–002277-22 (2018–06-25)).

**Table 1:** Demographics per cohort

Center		BBRC	GMC	CHUT
Tracer		[^18^F]flutemetamol	[^18^F]flutemetamol	[^18^F]florbetaben
*N*		68	107	75
Cohort Aβ + status (%)		AMYPAD PNHS 28.3	AMYPAD DPMS, PNHS and GMC 57.9*	EPAD LCS 12.0
Age (y)		63.3 ± 5.9	73.8 ± 8.9*	67.7 ± 7.5
Sex (F) (%)		51.2	49.5	66.6
Diagnosis (%)	CN^#^ MCI Dementia Other	98.7 1.3 0.0 0.0	28.0 51.4 16.8 3.7	100.0 0.0 0.0 0.0
Scanner		Siemens Biograph 64 mCT	Siemens Biograph 128 Edge mCT Flow and Siemens Biograph 128 Vision 600 Edge	Siemens Biograph 4 Emission Duo LSO and Siemens Truepoint HiRez
Scan duration		20 min (90–110 min p.i.)	20 min (90–110 min p.i.)	20 min (90–110 min p.i.)
Injected Dose		190.2 ± 14.3 MBq	179.4 ± 16.1 MBq	302.9 ± 23.3
Acquisition Type		List-mode	List-mode	List-mode
Reconstruction		OSEM, PSF, ToF	OSEM, PSF, ToF	OSEM, PSF
Reconstruction settings		3 iterations, 24 subsets, 5 mm gaussian filter	3 iterations, 24 subsets, 5 mm gaussian filter	3 iterations, 24 subsets, 5 mm gaussian filter

Values are depicted as mean ± SD, unless indicated otherwise. *CN* cognitively normal, *MCI* mild cognitive impairment, *AD* Alzheimer’s disease dementia, *DPMS* diagnostic and patient management study, *PNHS* prognostic and natural history study, *GMC* Geneva Memory Clinic cohort

* = *p* < 0.001 compared with BBRC FMM

^#^the CN group also includes individuals with subjective cognitive decline (SCD)

### Image acquisition

For [^18^F]flutemetamol, participants were injected with an average dose of 190.2 ± 14.3 MBq (BBRC cohort) or 179.4 ± 16.1 MBq (GMC cohort), and PET scans were acquired on a Siemens Biograph mCT or a Siemens Biograph Vision scanner. The remaining 75 participants from CHUT were injected with 302.9 ± 23.3 MBq [^18^F]florbetaben, and PET scans were acquired on a Siemens Biograph 4 Emission Duo LSO or Siemens Truepoint HiRez scanner (acquisition parameters can be found in Table 1). Prior to the PET scan, a non-diagnostic low-dose computed tomography (ldCT) scan was acquired on the same scanner for attenuation correction purposes. All scans were acquired in list-mode for 20 min, 90–110 min post radiotracer intravenous injection.

### List-mode processing

Reduced injected doses were simulated by manipulating the original PET list-mode data. List-mode data were delisted using vendor-provided reconstruction tools named “JSRecon” and “e7tools” (Siemens Molecular Imaging, Knoxville, USA). Across the entire 20-min acquisition window, from each one-second interval, true and random detection events were removed as a proportion of each injected dose level to represent a reduction in detection events across the entire acquisition time and therefore simulate a reduced injected dose, rather than simply a reduction in scan time. For example, at 50% of the originally injected dose, each one-second interval would have 50% of the true and random detection events removed. This method was used to generate the reduced-dose images corresponding to 75, 50, 25, 12.5, and 5% of the original injected dose.

### Post-processing

First, original, and reduced-dose list-mode data were reconstructed using an iterative 3D-ordered subset expectation maximisation (OSEM) algorithm with the default parameters in JSRecon. Point spread function (PSF) modelling, time-of-flight (ToF) corrections where available and attenuation correction using the corresponding ldCT scan were applied. Next, PET images were normalised to Montreal Neurological Institute (MNI) space using rPOP and the Statistical Parametric Mapping “Old Normalise Estimate & Write” function using a previously validated PET template consisting of [^18^F]flutemetamol, [^18^F]florbetaben and [^18^F]florbetapir data (SPM12, Wellcome Centre for Human Neuroimaging, London, UK) [30]. SUVRs were derived on a voxel-by-voxel basis using the cerebellar cortex as a reference region as defined by the Global Alzheimer’s Association Interactive Network (GAAIN) Centiloid project [4, 31–33]. A composite global cortical volume-of-interest (VOI), also from the GAAIN Centiloid project, and three VOIs known to show early Aβ accumulation, i.e., the precuneus, posterior cingulate cortex (PCC) and the orbitofrontal cortex (OFC) derived from the Desikan-Killiany atlas [34], were used as target regions for the quantitative analysis [35, 36]. To facilitate interpretability of the impact of dose reductions in absolute terms, we converted the global SUVR (from each tracer and cohort) to CL and will report this as additional outcome measure. CL values were pre-established using a validated pipeline at IXICO (London, UK) and regression lines were established for each tracer and cohort between the original, 100% SUVR and the CL values. These equations were subsequently applied to the reduced dose SUVRs, to convert these to CL values. The equations were the following: 107.65x-112.11 (BBRC), 121.21x-126.5 (GMC), and 124.65x-129.32 (CHUT).

### Visual assessment

Aβ status was determined by a trained nuclear medicine physician who visually assessed the original PET scans according to the respective tracer manufacturer’s reading guidelines [37]. To assess the effect of reduced doses on visual assessments as performed in a clinical setting, 32 scans from each tracer dataset were selected ([^18^F]flutemetamol: 16 Aβ-,16 Aβ + , range: -24 to 143CL, and [^18^F]florbetaben: 23 Aβ-, 9 Aβ + , range: -10 to 105CL) by a researcher who did not assess the scans, based upon the original full-dose visual assessment. From the 32 scans, 16 were considered grey-zone for [^18^F]flutemetamol and 15 for [^18^F]florbetaben, with grey-zone defined as having a CL between 12 and 50. [32, 38, 39]. Note, for [^18^F]flutemetamol there was an equal number of visually Aβ- and Aβ + grey-zone scans, while for [^18^F]florbetaben, the grey-zone group included 14 visually Aβ- scans and one Aβ + scan, as a result of the limited number of grey-zone and Aβ + scans in this dataset. For each of these scans, the original PET image (in counts) and the 75, 50, 25, 12.5 and 5% injected-dose images were assessed independently by two other trained readers for [^18^F]flutemetamol and one trained reader for [^18^F]florbetaben, without anatomical reference (i.e., MRI or CT scan) to replicate the most challenging clinical scenario. Scans were presented in random order and classified as either positive or negative and readers were blinded to the dose level and clinical information. In addition, a confidence score was provided based on a five-point scale (1 = very low confidence, 5 = very high confidence).

### Statistics

Between-cohort differences in age, proportion of males/females and Aβ + /Aβ- scans were investigated using Mann–Whitney U-tests and chi-square tests, respectively. Cortical SUVRs stratified by Aβ-status were visualised across dose levels and Mann–Whitney U-tests were used to verify whether SUVR differed significantly between Aβ-groups. Corresponding effect sizes were calculated using Hedge’s G. For SUVR, absolute and percentage differences were calculated between dose levels for all VOIs, while for CL only the absolute difference was calculated. In addition, receiver operating characteristic (ROC) analyses were performed to assess diagnostic accuracy and to determine the optimal sensitivity and specificity for distinguishing Aβ- from Aβ + scans. The area under the curve (AUC), Youden's index, sensitivity, and specificity were derived from the ROC analyses. The coefficient of variation (CoV) was calculated as a measure of variance (CoV = standard deviation/mean) and compared between dose levels for all VOIs. Bland–Altman analyses were used to assess potential bias between cortical SUVR of the original dose (i.e., 100%) and of each reduced dose level, and the presence of proportional bias was determined by fitting a regression line through the Bland–Altman plot.

To determine whether the visual assessment of the scan was affected by the reduction in dose, intra-reader agreement was assessed by comparing the reader’s assessment of the reduced dose images against their own read of the 100% images. Both the percentage agreement and Cohen’s kappa (κ) are reported. Agreement was assessed per tracer, per reader, for all scans and separately for the group of grey-zone (12 ≥ CL ≤ 50) and non-grey-zone scans (CL < 12 & CL > 50). In addition, the number of false positives (FP) and false negatives (FN) was calculated. Finally, Wilcoxon matched-pair signed rank tests were used to compare the average reader confidence scores between grey-zone and non-grey-zone scans and between dose levels.

## Results

### Demographics

Table 1 shows the demographics and acquisition parameters per cohort, stratified by tracer. With respect to [^18^F]flutemetamol, the vast majority of individuals from the BBRC cohort were cognitively normal (CN) (98.7%), compared with 20% from the GMC. Hence, as expected, participants from the BBRC cohort were significantly younger (*p* < 0.001), and the proportion of Aβ + individuals was significantly lower compared with the GMC (28.3 vs. 56.0%, respectively, *p* < 0.001).

### Quantitative discrimination between Aβ- and Aβ + scans across dose levels

**Fig. 1:**
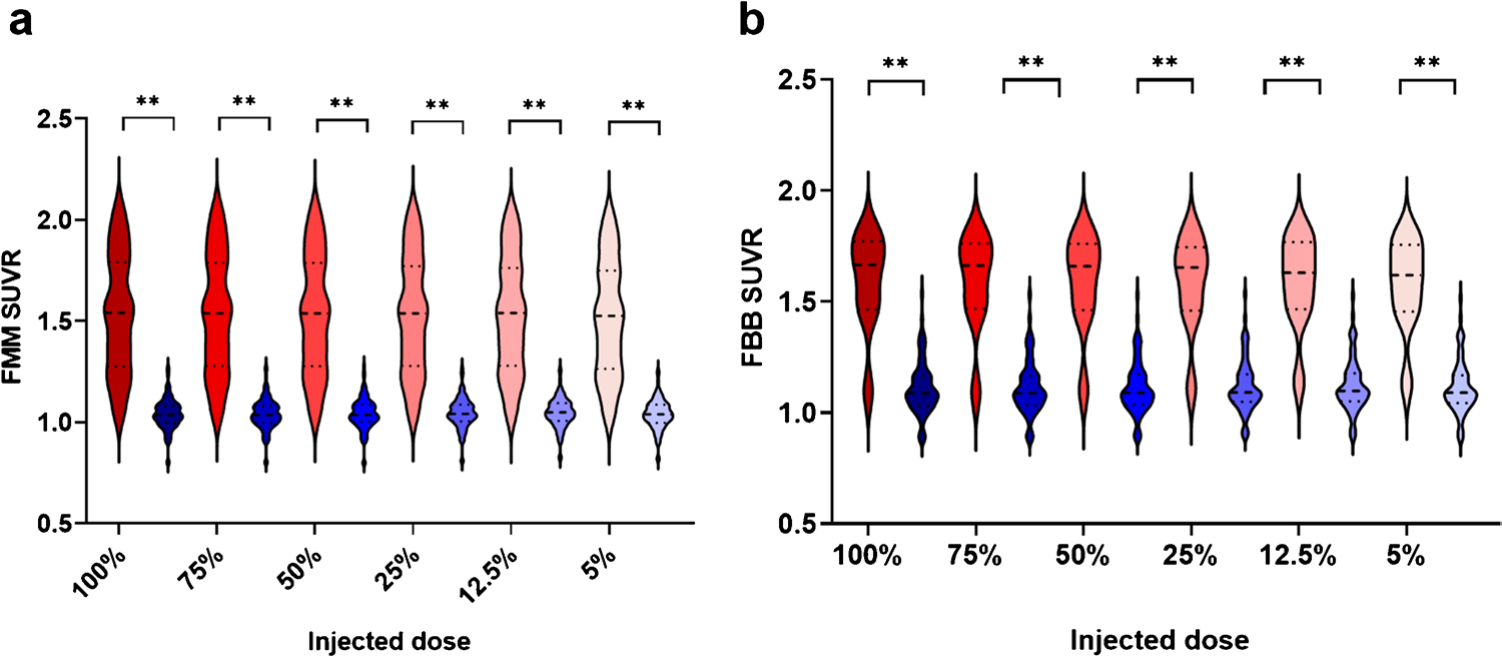
Cortical SUVRs for Aβ- and Aβ + participants across injected dose levels for (**a**) [^18^F]flutemetamol and (**b**) [^18^F]florbetaben. Boxes are colour-coded based upon the visual Aβ status of the scans, blue = Aβ- and red = Aβ + . Dashed lines correspond to the median and dotted lines to the quartiles. **p < 0.0001

For both tracers, cortical SUVRs differed significantly between Aβ- and Aβ + scans (Fig. 1a, b). For [^18^F]flutemetamol, the maximum difference in SUVR across dose levels was 1.92% for Aβ- individuals (absolute Δ in SUVR = 0.020 and in CL = 3.57) and 2.27% for Aβ + individuals (absolute Δ in SUVR = 0.035 and in CL = 4.62). CoV was considerably lower in the Aβ- compared with the Aβ + group, with a maximum difference of 0.70 percent point (pp) across dose levels (Table 2). For [^18^F]florbetaben, the maximum difference in SUVR across dose levels was 1.71% for Aβ- individuals (absolute Δ in SUVR = 0.019 and in CL = 2.43) and 1.70% for Aβ + individuals (absolute Δ in SUVR = 0.027 and in CL = 3.35). CoV was slightly lower for the Aβ- group and differed maximally 1.28 pp across dose levels (Table 2). For [^18^F]flutemetamol, the AUC was 0.96, and Youden's index ranged from 0.83 to 0.86 across dose levels. The sensitivity and specificity were consistently high, with sensitivity ≥ 90.79% and specificity ≥ 88.00% and the effect size was 2.41 across all dose levels, except for 5% where it was 2.14 (Table 3). For [^18^F]florbetaben, the AUC ranged from 0.94 to 0.96, and Youden's index ranged from 0.83 to 0.87 across dose levels. The sensitivity and specificity were high and stable across dose levels (≥ 94.03 and 88.89%, respectively) as well as the effect size which ranged from 2.61 to 2.93 (Table 3).

**Table 2:** Cortical SUVRs, Centiloid values and Coefficient of Variance across dose levels

[^18^F]flutemetamol	Aβ-negatives			Aβ-positives		
Dose (%)	SUVR	CoV (%)	Centiloid	SUVR	CoV (%)	Centiloid
100	1.040 ± 0.079	7.56	-1.49 ± 11.18	1.537 ± 0.294	19.15	74.88 ± 32.34
75	1.041 ± 0.079	7.56	-1.48 ± 11.13	1.536 ± 0.293	19.10	74.76 ± 32.20
50	1.042 ± 0.079	7.57	-1.47 ± 11.15	1.534 ± 0.292	19.02	74.42 ± 32.04
25	1.045 ± 0.077	7.36	-0.96 ± 10.95	1.531 ± 0.288	18.83	73.91 ± 31.54
12.5	1.047 ± 0.075	7.13	-0.48 ± 10.51	1.527 ± 0.286	18.74	73.57 ± 31.44
5	1.060 ± 0.084	7.93	2.08 ± 9.23	1.502 ± 0.281	18.69	70.26 ± 30.22
[^18^F]florbetaben	Aβ-negatives			Aβ-positives		
Dose (%)	SUVR	CoV (%)	Centiloid	SUVR	CoV (%)	Centiloid
100	1.114 ± 0.124	11.16	9.48 ± 15.49	1.588 ± 0.226	14.23	68.61 ± 28.16
75	1.114 ± 0.124	11.13	9.56 ± 15.46	1.585 ± 0.224	14.15	68.27 ± 27.96
50	1.117 ± 0.123	10.99	9.92 ± 15.31	1.584 ± 0.222	14.02	68.10 ± 27.68
25	1.120 ± 0.122	10.88	10.25 ± 15.18	1.581 ± 0.218	13.79	67.81 ± 27.18
12.5	1.121 ± 0.125	11.17	10.35 ± 15.60	1.587 ± 0.212	13.36	68.53 ± 26.42
5	1.133 ± 0.138	12.16	11.91 ± 17.20	1.561 ± 0.204	13.05	65.26 ± 25.43

Values are depicted as mean ± SD, unless indicated otherwise. *CoV* Coefficient of Variation (SD/mean)

**Table 3:** Effect sizes and ROC statistics for discriminating between Aβ groups

[^18^F]flutemetamol	Effect size (Hedge’s G)	AUC	Youden’s Index	Sensitivity (%)	Specificity (%)
Dose (%)					
100	2.41	0.96	0.85	96.91	88.00
75	2.41	0.96	0.85	95.88	88.00
50	2.41	0.96	0.85	96.91	88.00
25	2.41	0.96	0.86	94.85	90.67
12.5	2.41	0.96	0.86	96.91	89.93
5	2.14	0.96	0.83	90.79	91.78
[^18^F]florbetaben	Effect size (Hedge’s G)	AUC	Youdens Index	Sensitivity (%)	Specificity (%)
Dose (%)					
100	2.61	0.95	0.87	98.48	88.89
75	2.61	0.94	0.87	98.48	88.89
50	2.61	0.95	0.87	98.48	88.89
25	2.61	0.95	0.87	98.48	88.89
12.5	2.61	0.96	0.87	98.48	88.89
5	2.93	0.96	0.83	94.03	88.89

*AUC* area under the curve from the receiver operating curves (ROC)

For [^18^F]flutemetamol, VOI-based analyses showed that differences in mean SUVR across dose levels were a maximum of 3.72% for the precuneus, 2.10% for the PCC and 1.75% for the OFC (absolute Δ in SUVR ranged from 0.020 to 0.044). Differences in the CoV across dose levels were a maximum of 2.04 pp (Table 4). For [^18^F]florbetaben, differences in mean SUVR across dose levels were a maximum of 3.12% for the precuneus, 1.40% for the PCC and 0.35% for the OFC (absolute Δ in SUVR ranged from 0.004 to 0.035). Differences in CoV between dose levels were a maximum of 1.34 pp (Table 4). VOI-based results split by Aβ status were comparable and can be found in Table S1a, b and S2a, b.

**Table 4:** VOI-based SUVRs

[^18^F]flutemetamol	Precuneus		Posterior cingulate cortex		Orbitofrontal cortex	
Dose (%)	SUVR	CoV (%)	SUVR	CoV (%)	SUVR	CoV (%)
100	1.184 ± 0.316	26.65	1.331 ± 0.286	21.48	1.149 ± 0.295	25.65
75	1.185 ± 0.315	26.60	1.332 ± 0.285	21.38	1.149 ± 0.293	25.53
50	1.185 ± 0.312	26.36	1.332 ± 0.282	21.13	1.148 ± 0.290	25.24
25	1.188 ± 0.308	25.94	1.333 ± 0.277	20.77	1.147 ± 0.286	24.92
12.5	1.193 ± 0.308	25.79	1.337 ± 0.274	20.47	1.144 ± 0.282	24.64
5	1.228 ± 0.303	24.65	1.359 ± 0.264	19.44	1.164 ± 0.279	24.00
[18F]florbetaben	Precuneus		Posterior cingulate cortex		Orbitofrontal cortex	
Dose (%)	SUVR	CoV (%)	SUVR	CoV (%)	SUVR	CoV (%)
100	1.121 ± 0.199	17.72	1.289 ± 0.195	15.11	1.137 ± 0.214	18.82
75	1.123 ± 0.199	17.68	1.291 ± 0.194	15.02	1.137 ± 0.212	18.68
50	1.124 ± 0.198	17.58	1.290 ± 0.193	14.93	1.138 ± 0.212	18.59
25	1.131 ± 0.195	17.25	1.294 ± 0.188	14.54	1.138 ± 0.209	18.37
12.5	1.136 ± 0.195	17.16	1.295 ± 0.187	14.46	1.135 ± 0.209	18.38
5	1.156 ± 0.198	17.12	1.307 ± 0.199	15.25	1.139 ± 0.199	17.48

Values are depicted as mean ± SD, unless indicated otherwise. *VOI* volume of interest, *CoV* coefficient of variation (SD/mean)

### Agreement between cortical SUVRs of the original and reduced doses

**Fig. 2:**
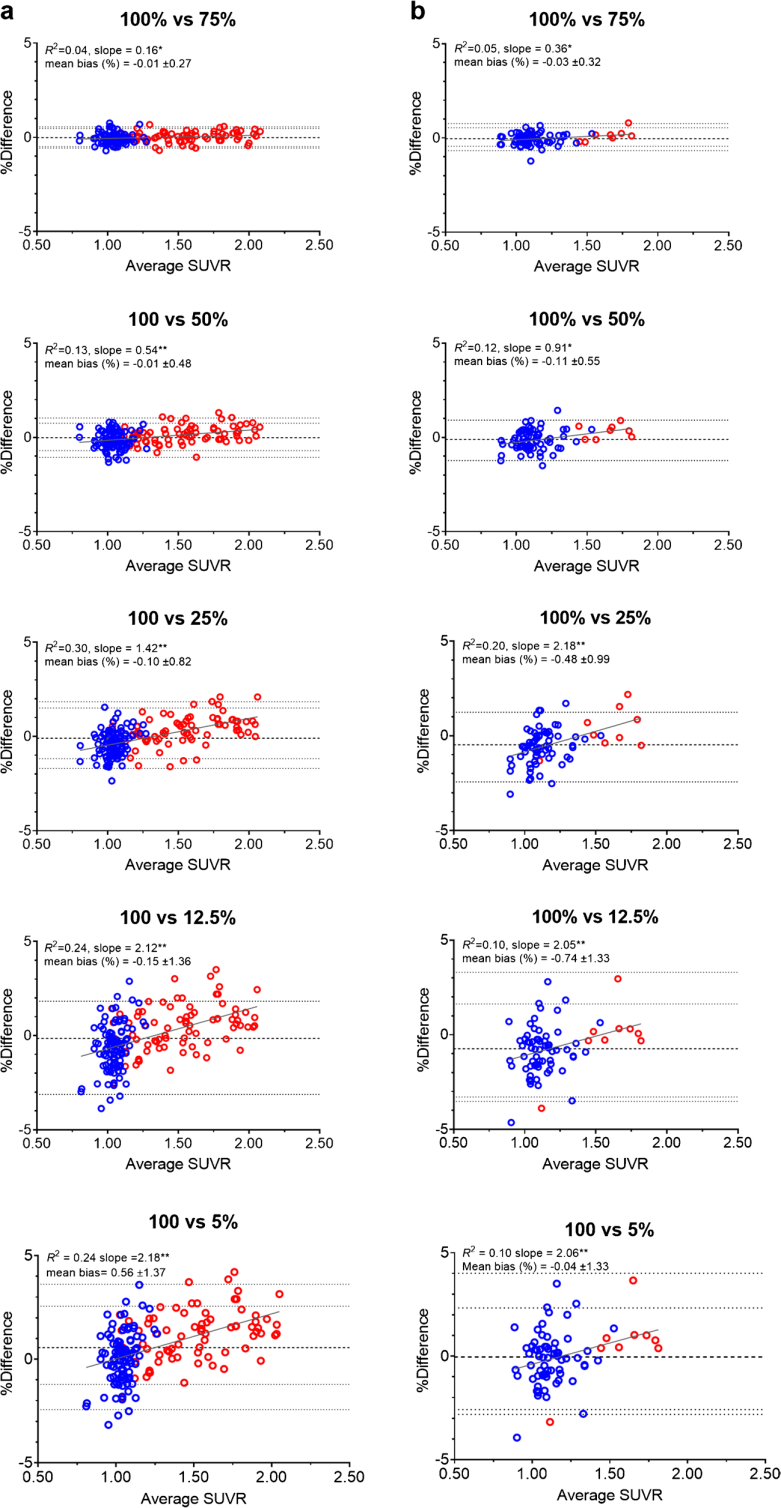
Agreement between cortical SUVR of the recommended (100%) and reduced injected dose levels for (**a**) [^18^F]flutemetamol and (**b**) [^18^F]florbetaben. Bland–Altman plots showing the relationship between cortical SUVR for 100% injected dose compared with reduced injected doses of 75, 50, 25, 12.5 and 5% from the original dose. Data-points are colour-coded based upon visual Aβ status, blue = Aβ- and red = Aβ + . The dashed horizontal line corresponds to the mean bias, the dotted horizontal lines correspond to the upper and lower limits of the 95% Limits of Agreement and the solid grey line to the linear regression of the BA data-points. * < 0.05, ** < 0.0001

For [^18^F]flutemetamol, Bland–Altman analyses showed that for injected dose levels of 75, 50, 25, 12.5 and 5%, mean bias in cortical SUVR compared with the original dose (100%) was -0.01 ± 0.27%, -0.01 ± 0.48%, -0.10 ± 0.82%, -0.15 ± 1.36% and 0.56 ± 1.37%. Lower injected doses showed an increased variability of the bias, as indicated by their SD (Fig. 2a). For [^18^F]florbetaben, mean bias in SUVR compared with the original dose was -0.03 ± 0.32%, -0.11 ± 0.55%, -0.48 ± 0.99%, -0.74 ± 1.33% and -0.04 ± 1.33% (for injected dose levels of 75, 50, 25, 12.5 and 5%, respectively), with increased variability for lower injected doses (Fig. 2b). For both tracers, bias was proportional to underlying levels of Aβ pathology across all dose levels.

### Intra-reader agreement on visual assessment

**Fig. 3:**
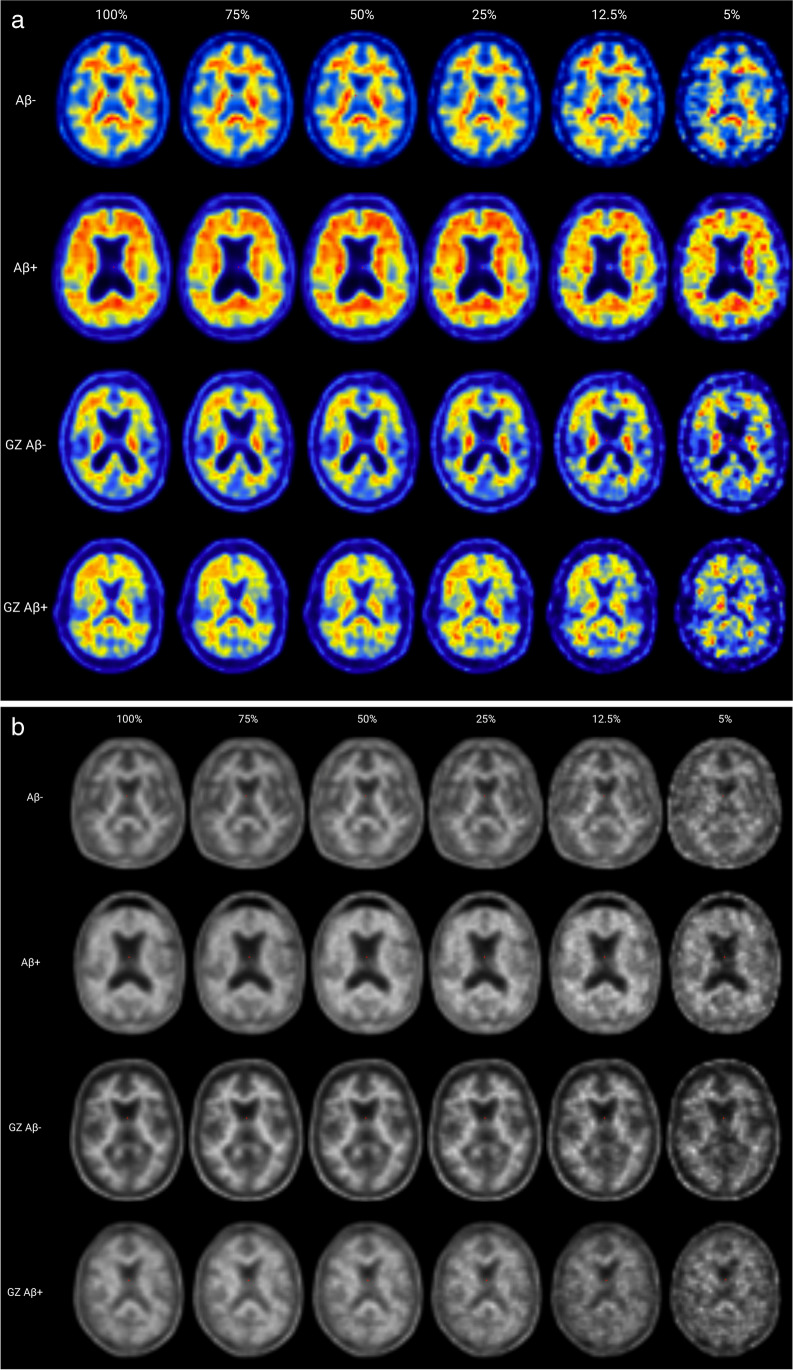
**a** Example image of Aβ- and Aβ + scans across injected dose levels for [^18^F]flutemetamol. Raw images of representative non-grey-zone and grey-zone Aβ- and Aβ + scans, to illustrate differences in visual interpretability between injected dose levels. Each image was visualized and assessed in accordance with manufacturer’s instructions for visual assessment of the scans. From top to bottom, rows show a visual Aβ- (CL = 3.1) and Aβ + (CL = 92.5) scan, a grey-zone (GZ), visual Aβ- (CL = 24.4) and a grey-zone (GZ), visual Aβ + scan (CL = 49.9). **b** Example image of Aβ- and Aβ + scans across injected dose levels for [^18^F]florbetaben. Raw images of representative non-grey-zone and grey-zone Aβ- and Aβ + scans, to illustrate differences in visual interpretability between injected dose levels. Each image was visualized and assessed in accordance with manufacturer’s instructions for visual assessment of the scans. From top to bottom, rows show a visual Aβ- (CL = -1.2) and Aβ + (CL = 97.1) scan, a grey-zone (GZ) Aβ- (CL = 18.3) and a grey-zone (GZ), visual Aβ + scan (CL = 23.2)

Figure 3a shows a representative [^18^F]flutemetamol image of two visually Aβ- (CL = 3.1, CL = 49.9) and two visually Aβ + scans (CL = 40.4, CL = 92.5). Figure 3b shows a representative [^18^F]florbetaben image of two visually Aβ- (CL = -1.2, CL = 18.3) and two visually Aβ + scans (CL = 23.2, CL = 97.1), to demonstrate the effect of reduced doses on visual interpretability of the scans.

**Fig. 4:**
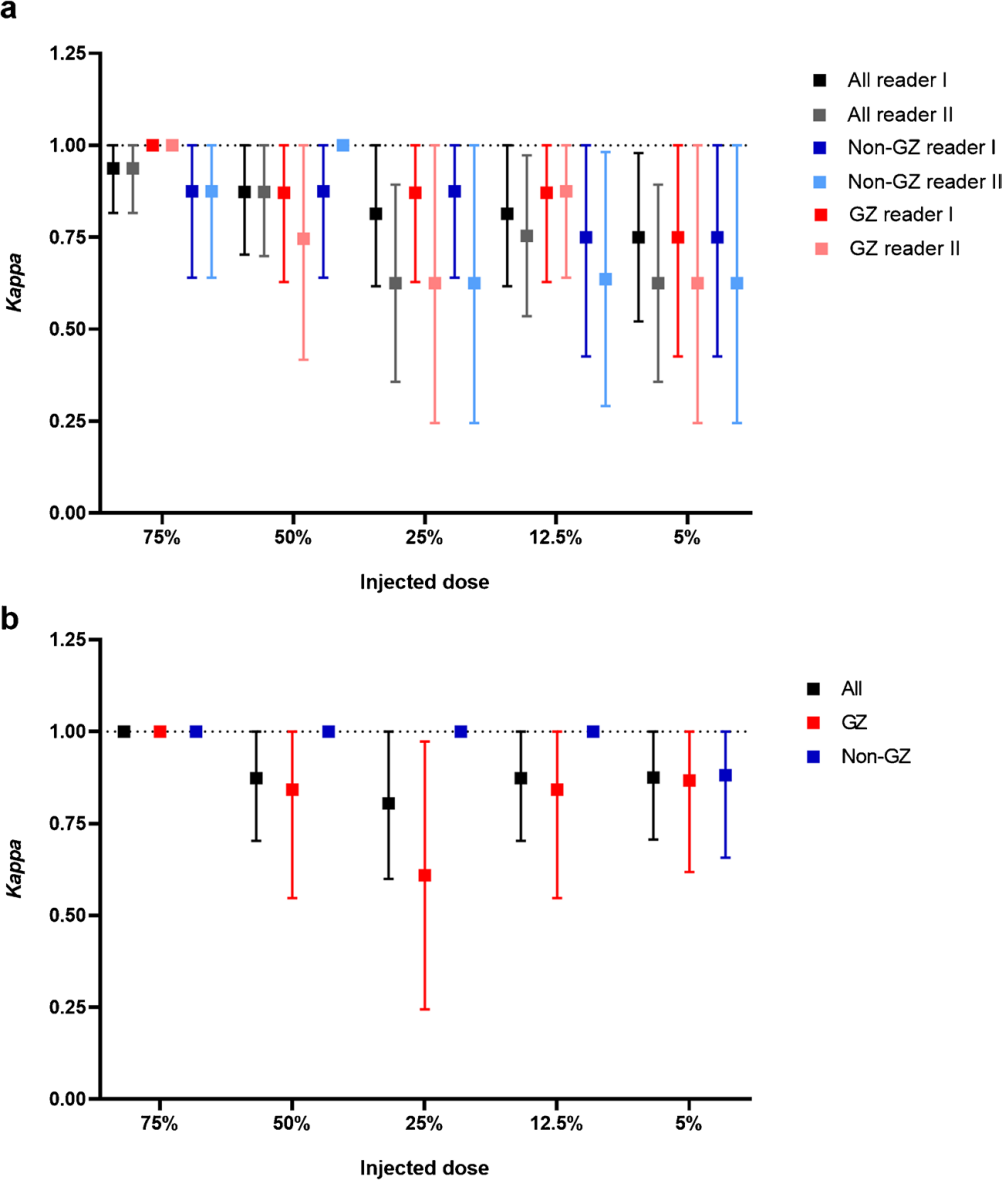
Intra-reader agreement between 100% and reduced dose images for (**a**) [^18^F]flutemetamol and (**b**) [^18^F]florbetaben. Kappas and confidence intervals across injected dose levels, for all scans (*N* = 32, black), and split for grey zone (*N* = 16 for [^18^F]flutemetamol and *N* = 15 for [^18^F]florbetaben, red) and non-grey-zone (*N* = 16 for [^18^F]flutemetamol and *N* = 17 for [^18^F]florbetaben, blue) scans. The dotted line indicates a kappa of 1, i.e., 100% intra-reader agreement

For [^18^F]flutemetamol, intra-reader agreement of all scans ranged from 81–97% (κ range: 0.63–0.94), with small differences between readers (Table 5). While a trend towards reduced agreement at lower dose levels was observed, no significant association was found between intra-reader agreement and dose levels, with the lowest agreement across all scans reported for the 25 and 5% dose levels. Furthermore, we did not find evidence for a difference in intra-reader agreement between grey-zone and non-grey-zone scans (Table 5, Fig. 4a). For [^18^F]florbetaben, intra-reader agreement of all scans ranged from 88–100% (κ range: 0.81–1.00). Again, agreement appeared slightly lower at lower injected doses, however, no significant relation between intra-reader agreement and dose levels was observed, and the lowest intra-reader agreement (i.e., 88%) was observed for the 25% dose level. For [^18^F]florbetaben, we did not observe differences in intra-reader agreement between grey-zone and non-grey-zone scans (Table 4, Fig. 4b), nor did we observe an association between the number of false positives (FP) or false negatives (FN) and dose levels for either tracer (Table S3).

**Table 5:** Intra-reader agreement between visual assessment of the 100% and reduced dose images

FMM—Reader I	Agreement (%)			Cohen’s κ		
Dose (%)	All	12 ≥ CL ≤ 50grey zone	CL < 12 & CL < 50	All	12 ≥ CL ≤ 50grey-zone	CL < 12 & CL > 50
75	97 (31/32)	100 (16/16)	94 (15/16)	0.94 ± 0.06	1.00 ± 0.00	0.88 ± 0.12
50	94 (30/32)	94 (15/16)	94 (15/16)	0.87 ± 0.09	0.87 ± 0.12	0.88 ± 0.12
25	94 (30/32)	94 (15/16)	94 (15/16)	0.87 ± 0.09	0.87 ± 0.12	0.88 ± 0.12
12.5	91 (29/32)	94 (15/16)	88 (14/16)	0.81 ± 0.10	0.87 ± 0.12	0.75 ± 0.17
5	88 (28/32)	88 (14/16)	88 (14/16)	0.75 ± 0.12	0.75 ± 0.17	0.75 ± 0.17
FMM—Reader II	Agreement (%)			Cohen’s κ		
Dose (%)	All	12 ≥ CL ≤ 50 grey zone	CL < 12 & CL < 50	All	12 ≥ CL ≤ 50 grey-zone	CL < 12 & CL > 50
75	97 (31/32)	100 (16/16)	94 (15/16)	0.94 ± 0.06	1.00 ± 0.00	0.88 ± 0.12
50	94 (30/32)	88 (14/16)	100 (16/16)	0.87 ± 0.09	0.75 ± 0.17	1.00 ± 0.00
25	81 (26/32)	81 (13/16)	81 (13/16)	0.63 ± 0.14	0.63 ± 0.19	0.63 ± 0.19
12.5	88 (28/32)	94 (15/16)	81 (13/16)	0.75 ± 0.11	0.88 ± 0.12	0.64 ± 0.18
5	81 (26/32)	81 (13/16)	81 (13/16)	0.63 ± 0.14	0.63 ± 0.19	0.63 ± 0.19
FBB—Reader I	Agreement (%)			Cohen’s κ		
Dose (%)	All	12 ≥ CL ≤ 50 grey zone	CL < 12 & CL < 50	All	12 ≥ CL ≤ 50 grey-zone	CL < 12 & CL > 50
75	100 (32/32)	100 (15/15)	100 (17/17)	1.00 ± 0.00	1.00 ± 0.00	1.00 ± 0.00
50	97 (31/32)	93 (14/15)	100 (17/17)	0.94 ± 0.06	0.87 ± 0.13	1.00 ± 0.00
25	88 (28/32)	73 (11/15)	100 (17/17)	0.81 ± 0.11	0.61 ± 0.19	1.00 ± 0.00
12.5	97 (31/32)	93 (14/15)	100 (17/17)	0.94 ± 0.06	0.84 ± 0.15	1.00 ± 0.00
5	94 (30/32)	93 (14/15)	94 (16/17)	0.88 ± 0.09	0.87 ± 0.13	0.88 ± 0.12

*FMM* [^18^F]flutemetamol, *FBB* [^18^F]florbetaben. All values are depicted as mean ± SE, unless indicated otherwise. *CL* Centiloid

At each dose level, the reader confidence was highly similar between grey-zone and non-grey-zone scans, and there was minimal to no degradation in reader confidence as dose was reduced (Table 6). A significant relationship between dose level and confidence scores was observed for the group consisting of all [^18^F]flutemetamol scans for reader II (*p* < 0.003) and for the group consisting of all [^18^F]florbetaben scans (*p* = 0.028) (Table 6).

**Table 6:** Reader confidence across dose levels

FMM—Reader I	Reader confidence		
Dose (%)	All	12 ≥ CL ≤ 50 grey-zone	CL < 12 & CL > 50
100	4.66 ± 0.61	4.78 ± 0.34	4.55 ± 0.72
75	4.69 ± 0.71	4.81 ± 0.66	4.58 ± 1.12
50	4.55 ± 0.59	4.67 ± 0.47	4.44 ± 0.66
25	4.74 ± 0.68	4.86 ± 0.55	4.63 ± 0.78
12.5	4.50 ± 0.95	4.61 ± 0.57	4.39 ± 1.11
5	4.47 ± 0.84	4.44 ± 0.96	4.50 ± 0.73
FMM—Reader II	Reader confidence		
Dose (%)	All	12 ≥ CL ≤ 50 grey-zone	CL < 12 & CL > 50
100	4.75 ± 0.57	4.63 ± 0.34	4.88 ± 0.72
75	4.66 ± 0.70	4.63 ± 0.66	4.69 ± 0.80
50	4.63 ± 0.66	4.50 ± 0.48	4.75 ± 0.78
25	4.45 ± 0.62	4.25 ± 0.50	4.67 ± 0.73
12.5	4.27 ± 1.05	4.13 ± 0.60	4.40 ± 1.11
5	4.18 ± 1.00	4.13 ± 1.02	4.25 ± 1.00
FBB—Reader I	Reader confidence		
Dose (%)	All	12 ≥ CL ≤ 50 grey-zone	CL < 12 & CL > 50
100	4.56 ± 0.56	4.26 ± 0.37	4.82 ± 0.69
75	4.69 ± 0.77	4.40 ± 0.58	4.94 ± 1.01
50	4.56 ± 0.65	4.20 ± 0.47	4.88 ± 0.76
25	4.41 ± 0.68	4.00 ± 0.63	4.76 ± 0.80
12.5	4.34 ± 1.02	4.00 ± 0.72	4.64 ± 1.16
5	4.21 ± 1.04	3.87 ± 1.30	4.52 ± 0.62

*FMM* [^18^F]flutemetamol, *FBB* [^18^F]florbetaben. Values are depicted as mean ± SD. *CL* Centiloid

## Discussion

Quantitative analyses showed that for both tracers, injecting only 5% of the original dose resulted in a maximum change of 3.72% in SUVR and 2.04 pp in its variability. The ability to quantitatively separate Aβ- from Aβ + scans was high (Hedge’s G > 2.0) across dose levels. Visual assessment of the scans showed that for both tracers and all dose levels, the intra-reader agreement was > 80% suggesting that scans with significant dose reductions could still be used for qualitative assessment of amyloid scans.

Quantitative analyses showed similar signal-to-noise ratios for SUVRs across dose levels, as shown by the effect sizes, apart from a small drop in effect size for [^18^F]flutemetamol at 5%. Furthermore, high AUC values and stable Youden’s Indices were reported across doses levels, demonstrating the feasibility of discriminating between Aβ-groups at lower doses for both tracers. These results demonstrate that injecting 5% of the original dose does not have a meaningful impact on the ability to quantitatively differentiate Aβ- from Aβ + scans. The maximum effect of reduced doses on cortical SUVR, CoV and CL was 2.27%, 0.70 pp and 4.62CL for [^18^F]flutemetamol and 1.71%, 1.28 pp and 3.35CL for [^18^F]florbetaben, at the 5% injected dose level. This magnitude of change is comparable to annual rates of change (i.e., 3.5–5.2CL) reported in Aβ + individuals [40], which implies that it could hinder separation of non-accumulators from true accumulators. At 12.5% injected dose, the absolute change in CL was only 1.31 and 0.87CL for [^18^F]flutemetamol and [^18^F]florbetaben, respectively. Furthermore, only at 5%, coregistration to the MNI template failed for five scans. These results agree to a certain extent with those reported by Herholz and colleagues for [^18^F]florbetapir, whom reported that the effect of reduced doses (i.e., 50, 20 and 10% of the original dose) on the mean cortical signal was minimal and no systematic bias was observed compared with the original dose [19]. However, their findings differed in that they reported a dose-dependent increase in CoV for lower injected doses, and in that their CoVs were considerably lower than ours [19]. The differences with the present study could be explained by a number of factors, first, their results correspond to a different amyloid tracer (i.e., [^18^F]florbetapir), second, their target ROI was slightly different from ours (comprising all cortical GM rather than the GAAIN cortical ROI), and they used a different scanner and reconstruction algorithm, which are factors known to affect the signal-to-noise ratio (SNR). Finally, Herholz and colleagues included list-mode datasets of only four participants, and used a bootstrapping procedure to generate a larger dataset, while our study included list-mode datasets from *N* = 175 and *N* = 75 participants for [^18^F]flutemetamol and [^18^F]florbetaben, respectively, contributing to the greater CoVs, and to a more realistic definition of Aβ-groups.

As smaller regions tend to have higher intrinsic noise levels than larger ones, we conducted the same quantitative analyses for three regions known to show early Aβ accumulation [35, 36]. For these smaller regions, the impact of injecting reduced doses resulted in a maximum change in SUVR of 3.72% at 5% and 0.76% at 12.5% injected dose. Vandenberghe and colleagues reported test–retest variability of [^18^F]flutemetamol cortical SUVR to be 1.5 ± 0.7%, using a cerebellar cortex reference region. At 5% injected dose, the maximum change in SUVR in the present study falls outside the reported test–retest variability, while at 12.5%, it falls within this range, suggesting that injecting 12.5% dose is still acceptable [18]. Moreover, based on previous research, it would be reasonable to assume that test–retest variability of smaller cortical regions would be similar or higher than 1.5 ± 0.7%, suggesting that the maximum change in SUVR reported here for 12.5% can be considered insignificant [41–43]. For [^18^F]florbetaben, maximum change in SUVR for these smaller regions was 3.12% at 5% injected dose and 1.34% at 12.5% injected dose. A previous study reported average cortical SUVR test–retest variability of [^18^F]florbetaben (using cerebellar cortex as reference region) CN participants to be 2.9% (range 0.1–9.0) [44]. This suggests that the change induced in SUVR at 12.5% injected dose (observed in the present study) would still be acceptable. Nonetheless, it should be noted that these test–retest studies are from 2010 and 2009 respectively, and test–retest variability may have improved for the currently used scanners. Bland–Altman analyses showed no systematic bias between original and reduced doses, although variability of the bias increased for lower injected doses. A bias proportional to underlying levels of Aβ pathology was observed for the lowest dose levels, which indicated that both the negative and the positive bias increased in magnitude.

Regarding the visual assessment of the [^18^F]flutemetamol scans, intra-reader agreement for all scans ranged from 81–97% with κ ranging from 0.63–0.94. Prior studies on intra-reader agreement for [^18^F]flutemetamol have reported intra-reader kappa values ranging from κ = 0.71–0.96, with great differences across study populations [45, 46]. Overall, our kappa values appear comparable to these previously reported values, except for the 25% and 5% dose level [^18^F]flutemetamol reader II results, that fall just outside this window (κ = 0.63). This suggests that level of experience from the reader may also play a role in the ability to assess reduced dose scans [47]. For [^18^F]florbetaben, intra-reader agreement for all scans ranged from 88–100% with κ ranging from 0.81–1.00. Two other studies that assessed intra-reader agreement using [^18^F]florbetaben reported an average κ = 0.78 in newly trained readers in a diverse study population and an agreement of 91–98% in CN participants and AD dementia patients [48, 49]. Our results are comparable to these previous studies, except for those in the 25% grey-zone group (agreement 73% and κ = 0.61) that fall outside this window. Overall, intra-reader agreement was considered adequate for both tracers and all dose levels even in the most challenging clinical cases and without anatomical reference [50]. It has been suggested that visual assessment of reduced dose scans might be more challenging for grey-zone cases [19], however, the present study did not find significant differences between grey-zone and non-grey-zone scans.

The present results suggest that reducing the injected dose to 12.5% for both tracers has limited effects on the quantitative and visual assessment of the scans, potentially enabling acquisition of additional PET scans within the same individual. A reduction below 12.5% is not recommended due to the aforementioned co-registration issues and meaningful quantitative effect on SUVR and CL. Note that our study assessed reconstructions from simulated reduced tracer doses rather than actual different tracer dose injections. However, differences between these methods are expected to be minor and are therefore unlikely to affect the overall conclusions of this study. This proof-of-concept study should thus be used as a guide in addition to a specific centre’s clinical or research circumstances when considering using reduced injected doses or to infer the use of reduced acquisition times from these results. Effects such as motion correction improvements and slight changes to tracer uptake for reduced acquisition times as well as changes in reconstruction algorithms for different scanners should be taken into account.

## Conclusion

Our study shows that for both [^18^F]flutemetamol and [^18^F]florbetaben, adequate quantitative and qualitative assessments can be obtained with injected doses of 12.5% of what is typically recommended today. Reductions in injected dose will lead to a significant reduction in radiation exposure, reduced costs of the PET scan and potentially enable acquisition of more research scans of the same individual. Ultimately, decisions to reduce the injected dose should be made considering the specific clinical or research circumstances and take these findings into account.

### Supplementary Information

Below is the link to the electronic supplementary material.

Supplementary file1 (DOCX 28 KB)

## Data Availability

The data generated as part of this study can be made available upon reasonable request. Any requests regarding the list-mode data received from Barcelonaβeta Brain Research Center, Geneva University Hospital or Centre Hospitalier Universitaire de Toulouse can be directed towards the corresponding authors.
