# Supplementary material for: Impact of simulated reduced injected dose on the assessment of amyloid PET scans
Source: Eur J Nucl Med Mol Imaging. 2023 Oct 28;51(3):734–48. doi: 10.1007/s00259-023-06481-0 (PMC10796642; doi:10.1007/s00259-023-06481-0)
Supplement: Supplementary file 1 — Supplementary file1 (DOCX 28 KB) [file 259_2023_6481_MOESM1_ESM.docx]

**Supplementary Materials**

**Table 1a. [^18^F]Flutemetamol VOI-based SUVRs for Aβ- scans**

| **Dose (%)** | **Precuneus** | | **Posterior cingulate cortex** | | **Orbitofrontal cortex** | |
| --- | --- | --- | --- | --- | --- | --- |
|  | **SUVR** | **CoV (%)** | **SUVR** | **CoV (%)** | **SUVR** | **CoV (%)** |
| 100 | 1.011 ± 0.140 | 13.84 | 1.174 ± 0.136 | 11.58 | 0.974 ± 0.084 | 8.66 |
| 75 | 1.012 ± 0.139 | 13.78 | 1.174 ± 0.135 | 11.48 | 0.974 ± 0.084 | 8.62 |
| 50 | 1.015 ± 0.139 | 13.75 | 1.176 ± 0.134 | 11.43 | 0.976 ± 0.083 | 8.48 |
| 25 | 1.020 ± 0.138 | 13.49 | 1.180 ± 0.131 | 11.10 | 0.977 ± 0.082 | 8.42 |
| 12.5 | 1.025 ± 0.135 | 13.19 | 1.187 ± 0.132 | 11.13 | 0.977 ± 0.079 | 8.04 |
| 5 | 1.031 ± 0.132 | 13.07 | 1.194 ± 0.130 | 11.04 | 0.979 ± 0.082 | 8.09 |

**Table 1b. [^18^F]Florbetaben VOI-based SUVRs for Aβ- scans**

| **Dose (%)** | **Precuneus** | | **Posterior cingulate cortex** | | **Orbitofrontal cortex** | |
| --- | --- | --- | --- | --- | --- | --- |
|  | **SUVR** | **CoV (%)** | **SUVR** | **CoV (%)** | **SUVR** | **CoV (%)** |
| 100 | 1.011 ± 0.140 | 13.85 | 1.244 ± 0.138 | 11.11 | 1.072 ± 0.128 | 11.99 |
| 75 | 1.012 ± 0.140 | 13.79 | 1.245 ± 0.138 | 11.10 | 1.073 ± 0.129 | 11.99 |
| 50 | 1.014 ± 0.140 | 13.76 | 1.246 ± 0.139 | 11.14 | 1.074 ± 0.128 | 11.91 |
| 25 | 1.020 ± 0.138 | 13.51 | 1.252 ± 0.138 | 10.99 | 1.082 ± 0.126 | 11.68 |
| 12.5 | 1.025 ± 0.135 | 13.21 | 1.254 ± 0.142 | 11.31 | 1.087 ± 0.128 | 11.79 |
| 5 | 1.036 ± 0.127 | 13.34 | 1.266 ± 0.135 | 11.44 | 1.095 ± 0.133 | 12.01 |

**Table 2a. [^18^F]Flutemetamol VOI-based SUVRs for Aβ+ scans**

| **Dose (%)** | **Precuneus** | | **Posterior cingulate cortex** | | **Orbitofrontal cortex** | |
| --- | --- | --- | --- | --- | --- | --- |
|  | **SUVR** | **CoV (%)** | **SUVR** | **CoV (%)** | **SUVR** | **CoV (%)** |
| 100 | 1.553 ± 0.271 | 17.44 | 1.669 ± 0.229 | 13.73 | 1.531 ± 0.219 | 14.31 |
| 75 | 1.552 ± 0.270 | 17.41 | 1.669 ± 0.227 | 13.60 | 1.528 ± 0.217 | 14.23 |
| 50 | 1.549 ± 0.269 | 17.36 | 1.665 ± 0.222 | 13.35 | 1.524 ± 0.215 | 14.13 |
| 25 | 1.547 ± 0.267 | 17.28 | 1.660 ± 0.220 | 13.23 | 1.518 ± 0.211 | 13.91 |
| 12.5 | 1.552 ± 0.267 | 17.23 | 1.659 ± 0.218 | 13.15 | 1.510 ± 0.211 | 13.97 |
| 5 | 1.597 ± 0.261 | 17.89 | 1.650 ± 0.207 | 12.97 | 1.501 ± 0.203 | 13.90 |

**Table 2a. [^18^F]Florbetaben VOI-based SUVRs for Aβ+ scans**

| **Dose (%)** | **Precuneus** | | **Posterior cingulate cortex** | | **Orbitofrontal cortex** | |
| --- | --- | --- | --- | --- | --- | --- |
|  | **SUVR** | **CoV (%)** | **SUVR** | **CoV (%)** | **SUVR** | **CoV (%)** |
| 100 | 1.489 ± 0.244 | 16.36 | 1.625 ± 0.229 | 14.11 | 1.561 ± 0.199 | 12.77 |
| 75 | 1.489 ± 0.243 | 16.31 | 1.621 ± 0.229 | 14.10 | 1.556 ± 0.199 | 12.82 |
| 50 | 1.490 ± 0.239 | 16.05 | 1.616 ± 0.226 | 13.97 | 1.555 ± 0.200 | 12.85 |
| 25 | 1.491 ± 0.238 | 15.99 | 1.607 ± 0.221 | 13.76 | 1.548 ± 0.191 | 12.31 |
| 12.5 | 1.497 ± 0.227 | 15.18 | 1.603 ± 0.201 | 12.54 | 1.548 ± 0.182 | 12.76 |
| 5 | 1.504 ± 0.222 | 14.96 | 1.587 ± 0.231 | 12.44 | 1.521 ± 0.180 | 12.66 |

**Table 3. False positive and false negative assessment across different dose levels**

| **FMM - Reader I** | **All** | | | **12 ≥CL≤ 50 grey-zone** | | | | **CL<12 & CL>50** | | | |
| --- | --- | --- | --- | --- | --- | --- | --- | --- | --- | --- | --- |
| **Dose (%)** | **FP** | **FN** | | **FP** | | **FN** | | **FP** | | **FN** | |
| 75 | 1 | 0 | | 0 | | 0 | | 1 | | 0 | |
| 50 | 0 | 2 | | 0 | | 1 | | 0 | | 1 | |
| 25 | 0 | 2 | | 0 | | 1 | | 0 | | 1 | |
| 12.5 | 1 | 2 | | 0 | | 1 | | 1 | | 1 | |
| 5 | 2 | 2 | | 1 | | 1 | | 1 | | 1 | |
| **FMM - Reader II** | **All** | | | | **12 ≥CL≤ 50 grey-zone** | | | | **CL<12 & CL>50** | | |
| **Dose (%)** | **FP** | | **FN** | | **FP** | | **FN** | | **FP** | | **FN** |
| 75 | 1 | 0 | | 0 | | 0 | | 1 | | 0 | |
| 50 | 2 | 0 | | 2 | | 0 | | 0 | | 0 | |
| 25 | 4 | 2 | | 2 | | 1 | | 2 | | 1 | |
| 12.5 | 4 | 0 | | 1 | | 0 | | 3 | | 0 | |
| 5 | 4 | 2 | | 1 | | 1 | | 3 | | 1 | |
| **FBB - Reader I** | **All** | | | | **12 ≥CL≤ 50 grey-zone** | | | | **CL<12 & CL>50** | | |
| **Dose (%)** | **FP** | | **FN** | | **FP** | | **FN** | | **FP** | | **FN** |
| 75 | 0 | | 0 | | 0 | | 0 | | 0 | | 0 |
| 50 | 0 | | 1 | | 0 | | 1 | | 0 | | 0 |
| 25 | 3 | | 1 | | 3 | | 1 | | 0 | | 0 |
| 12.5 | 0 | | 1 | | 0 | | 1 | | 0 | | 0 |
| 5 | 1 | | 1 | | 1 | | 0 | | 0 | | 1 |

FMM = [^18^F]flutemetamol, FBB = [^18^F]florbetaben, FP=false positive, FN=false negative, CL = Centiloid
